# CD83: Activation Marker for Antigen Presenting Cells and Its Therapeutic Potential

**DOI:** 10.3389/fimmu.2019.01312

**Published:** 2019-06-07

**Authors:** Ziduo Li, Xinsheng Ju, Pablo A. Silveira, Edward Abadir, Wei-Hsun Hsu, Derek N. J. Hart, Georgina J. Clark

**Affiliations:** ^1^Dendritic Cell Research, ANZAC Research Institute, Sydney, NSW, Australia; ^2^Sydney Medical School, The University of Sydney, Sydney, NSW, Australia

**Keywords:** CD83, antigen presenting cells, immune suppression, therapeutic antibody, transplantation

## Abstract

CD83 is a member of the immunoglobulin (Ig) superfamily and is expressed in membrane bound or soluble forms. Membrane CD83 (mCD83) can be detected on a variety of activated immune cells, although it is most highly and stably expressed by mature dendritic cells (DC). mCD83 regulates maturation, activation and homeostasis. Soluble CD83 (sCD83), which is elevated in the serum of patients with autoimmune disease and some hematological malignancies is reported to have an immune suppressive function. While CD83 is emerging as a promising immune modulator with therapeutic potential, some important aspects such as its ligand/s, intracellular signaling pathways and modulators of its expression are unclear. In this review we discuss the recent biological findings and the potential clinical value of CD83 based therapeutics in various conditions including autoimmune disease, graft-vs.-host disease, transplantation and hematological malignancies.

## Introduction

The immune system's primary function is to protect the host from foreign pathogens, but its dysregulation can lead to serious illness or even death. For instance, failure of immunological tolerance has the potential to cause autoimmune diseases (1). In the transplantation setting, uncontrolled allogeneic immune responses leads to donor organ rejection or graft-vs.-host disease (GVHD) in which grafted T-cells respond to host tissue antigens presented by activated donor or host antigen-presenting cells (APC) (2). Current approaches to prevent or treat these diseases conventionally include non-specific immune suppression agents such as steroids or cyclosporin. However, these agents compromise the patient's immune function against pathogens and malignancy (3). There is a need for selective immune suppressive agents targeting specific inflammatory cells that prevent undesirable immune responses but preserve beneficial responses against infection and cancer.

Expression of membrane-bound (m)CD83 on the surface of activated dendritic cells (DC) and other APC make it an attractive therapeutic target, for achieving selective immune suppression. Anti-CD83 specific antibodies with the ability to deplete CD83^+^ cells have shown efficacy in the treatment of pre-clinical models of GVHD without significantly affecting viral or tumor specific memory T-cell responses (Table 1). Alternatively, recombinant soluble extracellular CD83 constructs (rsCD83) mimicking the natural soluble (s)CD83 variant have demonstrated potent immunosuppressive properties in animal models of autoimmune disease and transplantation (Table 1). In this article, we review the most recent literature updating our understanding of CD83 biology and discuss the value of applications using anti-CD83 antibodies or sCD83 in mediating immune suppression or targeting CD83^+^ malignancies.

**Table 1:** Therapeutic applications of CD83.

**Therapeutic**	**Indication**	**Potential mechanism**
Anti-CD83 antibody	Diagnostic mature DC marker	CD83 upregulation when DC are activated (4–8)
	Therapeutic target for GVHD	Depletion of mature CD83^+^ DC (9, 10)
	Therapeutic target for GCA	Depletion of mature CD83^+^ DC in GCA arteries (11)
Anti-CD83 antibody/antibody drug conjugates	Biomarker and therapeutic HL target	Depletion of CD83^+^ malignant cells; neutralizing peripheral sCD83 (12)
Serum sCD83	Biomarker in HL, chronic lymphocytic leukemia, and mantle cell lymphoma	Serum sCD83 shed from CD83^+^ malignant cells (12–15)
rsCD83	**Immunosuppressive agent in solid organ transplant rejection**	
	Skin transplant	Inhibition of recipient T-cell proliferation and IL-2, IFN-γ production (16)
	Kidney transplant	Tolerogenic DC generation; induction of IDO (17)
		Reduced number of infiltrating T-cells and monocytes and lower levels of inflammatory cytokines in graft (18)
	Corneal transplant	Regulatory T-cell induction mediated by IDO and TGF-β (19)
	Cardiac transplant	Attenuating DC maturation and function, such as down modulating MHC-II expression and reducing the DC allogeneic stimulatory capacity (20)
	**Immunosuppressive agent in autoimmune diseases**	
	MS (EAE)	Reduced T-cell proliferation and production of IFN-γ (21)
	Inflammatory Bowel Disease	Induced tolerogenic IDO^+^ DC (22)
	Autoimmune Uveitis (EAU)	NK cells reduced and the expression of CD11b and CD83 in NK cells decreased (23)
		Induced tolerogenic IDO^+^ DC by decreased expression of co-stimulatory molecules and hampered DC calcium response (17)
	Systemic Lupus Erythematosus	Reduction of splenic and peripheral IgG-secreting cells and peripheral T-cells (24)
	Rheumatoid arthritis	Reduced arthritis by increasing Treg numbers in an IDO and TGF-β dependent manner (25)

## Physiological Characteristics of CD83

### CD83 Structure and Expression

CD83 is a member of the immunoglobulin (Ig) superfamily. The human gene maps to Chromosome 6p23 and consists of 5 exons: exon 1 encoding the leader sequence, exons 2–3 the extracellular domain, exon 4 the transmembrane domain and exon 5 the intracellular domain (26). Similar gene organization is found in other mammals (27). The human CD83 protein comprises of 205 amino acids with extensive glycosylation resulting in a molecular weight of 45 kD. The mouse protein shows 63% similarity but is smaller (196 amino acids) due to the absence of an eleven amino acid sequence within the extracellular region (28, 29).

In mammals, fish and birds, mCD83 is recognized as an activation marker on the surface of immune cells (30). In humans and mice, the highest and most stable expression is found on activated DC from various tissues, including plasmacytoid and myeloid subsets (31–33). Nevertheless, mCD83 is found on the surface of other activated hematopoietic cells, including B-cells (34–36), macrophages, monocytes (37), neutrophils (38) and NK cells (39). In germinal centers, CD83 is expressed on B-cell centrocytes within the light zone undergoing selection and Ig class switching (40). mCD83 is only found on minor proportions of non-regulatory human T-cells that have engaged with APC, with surface expression primarily due to trogocytosis (35). mCD83 was not detected on the surface of human natural regulatory CD4^+^ T-cells (Treg) (35) but could be detected on induced or expanded Treg (41, 42). In mice, natural CD4^+^ Treg show high levels of CD83 promoter activity, and upon activation rapidly express mCD83 on their surface (35, 43, 44). mCD83 is also present on non-hematopoietic cortical thymic epithelial cells (TEC) in mice (45, 46), which is yet to be examined in humans.

Intracellular preformed CD83 protein is detected in a wide range of immune cells, including immature DC, monocytes, macrophages, natural-killer (NK) cells and lymphocytes (35). CD83 is rapidly transported to the surface from golgi and recycling endosome pools in DC, macrophages, monocytes and B-cells upon TLR or TNF engagement (37, 47). CD40/CD40L and BCR-ligation induces mCD83 in B-cells (48). Detailed analysis of the human CD83 gene promoter found SP-1 and NF-κB sites were critical for induction of the gene (49), with interferon regulatory factor-1, -2, -5 and NF-κB-p50, -p65, and -cRel involved in regulating CD83 expression in DC (50). The post-translation modulation of CD83 comprises of a golgi transport related protein, GRASP55, which binds to the CD83 C-terminal TELV-motif and plays a role in CD83 glycosylation (51).

sCD83 can be detected at low levels in healthy human sera but is elevated in the sera of patients with hematopoietic malignancies or autoimmune diseases (12–14, 52–55). Similarly, low to undetectable levels are found in the sera of healthy mice, which is elevated during pregnancy (56) or induction of autoimmunity (57). Culturing experiments determined that most sCD83 is produced by activated B-cells and DC (52, 56), as well as Treg in mice (43). The sequence of natural sCD83 in both species remains unconfirmed and as a result, it is not clear whether the product derives from cleavage of the extracellular portion of mCD83, CD83 splice variants (26), or both. The abundant amount of sCD83 produced by the Hodgkin derived cell line KM-H2, which only express the full-length CD83 transcript, supports cleavage (35); whereas detection of sCD83 in supernatants of cell lines transfected with certain CD83 splice variants supports the alternative mechanism (26).

### CD83 Ligands

CD83 forms homodimers in prokaryotic expression systems (29) confirmed in protein crystal structure analysis (58). Strong structural similarities between CD83 and B7 family members were revealed, suggesting that, like B7 family members, CD83 could exert its immunological activity by either homotypic or heterotypic interactions with a ligand (58). rsCD83 constructs bind to the surface of DC (59), B-cells (60), and monocytes (61), cells reported to express CD83 themselves. Similar constructs bound a CD83 transfected but not the wild-type Chinese hamster ovary cell line and failed to bind DC with a CD83 knock-down (62). This indicated the potential for homotypic binding of mCD83 in trans to mCD83 on other cells, but to date, investigators have failed to demonstrate a clear biophysical interaction. These studies do not preclude the possibility that CD83 binds to other ligands as predicted by structural analysis. Indeed, human rsCD83 has been shown to bind myeloid differentiation factor-2 (MD-2), a co-receptor associated with the TLR4 signaling complex, on monocytes (63).

### mCD83 Function

The important role of mCD83 in T-cell development became evident in CD83 knockout mice, which exhibit a severe reduction of CD4^+^ T-cells (45). This phenotype was intrinsic to the non-hematopoietic compartment and could be reversed by intra-thymic injection of wild-type TEC. The underlying mechanism by which mCD83 controls T-cell selection by TEC was attributed to its transmembrane region (64), which binds, and functionally inhibits, the membrane-associated RING-CH8 (MARCH-8) ubiquitin ligase (46, 65). Since MARCH-8 is responsible for the internalization and degradation of surface MHC class II (MHC-II) through ubiquitination, CD83 expression by cortical TEC stabilizes MHC-II on their surface permitting positive CD4^+^ T-cell selection. mCD83 similarly promotes upregulation of surface MHC-II and CD86 on activated APC including DC and B-cells through transmembrane regulation of the haematopoietically-restricted ubiquitin ligase MARCH-1 (64).

Despite being present on the surface of activated APC and causing upregulation of MHC-II and CD86 required for T-cell activation, the outcomes of signaling through surface mCD83 appears to lead to suppressed or regulatory functions in various immune cell populations. For DC, engagement of mCD83 with antibody or homotypic binding with CD83 expressing cell lines *in vitro* or transgenic CD83 expression by non-hematopoietic cells *in vivo* reduced their capacity to mature and secrete pro-inflammatory cytokines, a feature dependent on the MAPK signaling pathway (62). On the other hand, mice with a conditional knockout of CD83 in DC exhibited increased susceptibility to severe colitis, further indicative of a role for CD83 in DC regulation.

CD83 expression by mouse B or T-cells was shown to increase their longevity *in vivo* (66). However, transgenic overexpression of CD83 in mouse B-cells resulted in inhibitory function, as demonstrated by a decreased capacity to proliferate, class-switch and secrete Ig upon immunization (despite increased surface MHC-II and CD86 levels) as well as augmented secretion of the immunoregulatory cytokine IL-10 by marginal zone B-cells (67). Treating mice with anti-CD83 antibodies significantly augmented their IgG1 responses to T-cell independent antigens, which was underpinned by increased marginal zone B-cell isotype switching (68). Ablating CD83 expression conditionally in B-cells did not result in major changes to their response to antigen, though some changes were noted in germinal center composition and IgE class-switching (69). So far, little is known about CD83 function in human B-cells. However, targeting them *in vivo* with an anti-CD83 monoclonal antibody (mAb) in a human PBMC xenograft model inhibited B-cell responses to specific antigens without causing pan B-cell depletion (70).

In mice, CD83 expression is associated with regulatory function in T-cells. Using reporter mice, CD83 expression was associated with T-cells which mediate Treg-like functions *in vitro* and *in vivo* (43). Transduction of CD83 into mouse CD4^+^ CD25^−^ naïve T-cells imparted them with suppressive capabilities comparable to naturally occurring Treg including prevention of experimental autoimmune encephalomyelitis (EAE) in a mouse model (71). While expression of mCD83 on Treg could act in trans to downregulate the function of DC expressing mCD83, the molecule was shown to have essential intrinsic function in Treg differentiation and retention of their regulatory phenotype (42). In humans, continuous expression of CD83 on activated human CD4^+^ T-cells is indicative of their differentiation into induced Treg (41).

### sCD83 Function

To evaluate the potential function of sCD83, several studies have used rsCD83 constructs consisting of the human or mouse CD83 extracellular domain fused to an Ig Fc chain or a polyhistidine tag (4, 21, 26, 59, 62, 72–76). These all showed similar immune suppressive properties compared to control constructs, inhibiting human monocyte differentiation into DC (72, 76), changing the DC cytoskeleton (75), preventing DC maturation (59, 62), and reducing DC-mediated T-cell proliferation (4).

The ligand of sCD83 and how it exerts its immune inhibitory function is under investigation. Homotypic interaction of rsCD83 with mCD83 on DC blocks the production of inflammatory cytokines monocyte chemoattractant protein-1 and IL-12p40 through MAPK signaling (62). Another study showed that rsCD83 binding to DC suppressed f-actin mediated calcium signaling, preventing co-localization of ORAI1 and mitochondria at the DC-T-cell synapse (57). Binding of rsCD83 to the TLR4/MD-2 complex on monocytes induced anti-inflammatory mediators, such as indoleamine 2,3-dioxygenase (IDO), IL-10, and PGE2 in a COX-2-dependent manner, leading to inhibition of T-cell proliferation and IL-2 secretion (63, 72). The increased generation of IDO and TGF-β by rsCD83 leads to the induction of Treg and allograft tolerance, which was confirmed in mouse kidney or corneal transplant models (17, 19).

## Translation of CD83 into the Clinic

### CD83 as a DC Activation Marker and Viral Infection Target

mCD83 is an informative DC maturation marker (77, 78) and has been used in clinical trials of solid organ transplant rejection (clinicaltrials.gov, NCT01678937), DC vaccination for the treatment of melanoma (clinicaltrials.gov, NCT01425749) and acute myeloid leukemia (5) or as an inflammatory indicator for novel psoriasis therapy (clinicaltrials.gov, NCT01736696). CD83^+^ DC are reported to have prognostic value as an inverse correlate of gastric cancer outcomes (6).

Viruses have evolved a number of strategies to subvert host immunity including the targeting of CD83 on APC. Immature DC infected with Herpes-Simplex virus-1 (HSV-1) failed to express mCD83 during maturation (79) and the virus contributed to rapid downregulation of mCD83 on mature DC (7). Interestingly, HSV-1 secretes soluble factors, such as L particles, that interfere with CD83 expression on HSV-1-negative (uninfected) bystander DC (80). Similar observations of virus induced DC downregulation of mCD83 were found with varicella-zoster (81) and cytomegalovirus infection (4, 82). The latter downregulates mCD83 on mature DC via major immediate early viral effector protein IE2 and induces sCD83 secretion (82). In contrast, Epstein-Barr virus produces latent membrane protein-1, which induces rapid upregulation of mCD83 and a strong immune response against infected B-cells, establishing viral latency (8).

### sCD83 in Autoimmune Disorders and Solid Organ Transplant Rejection

**Figure 1:**
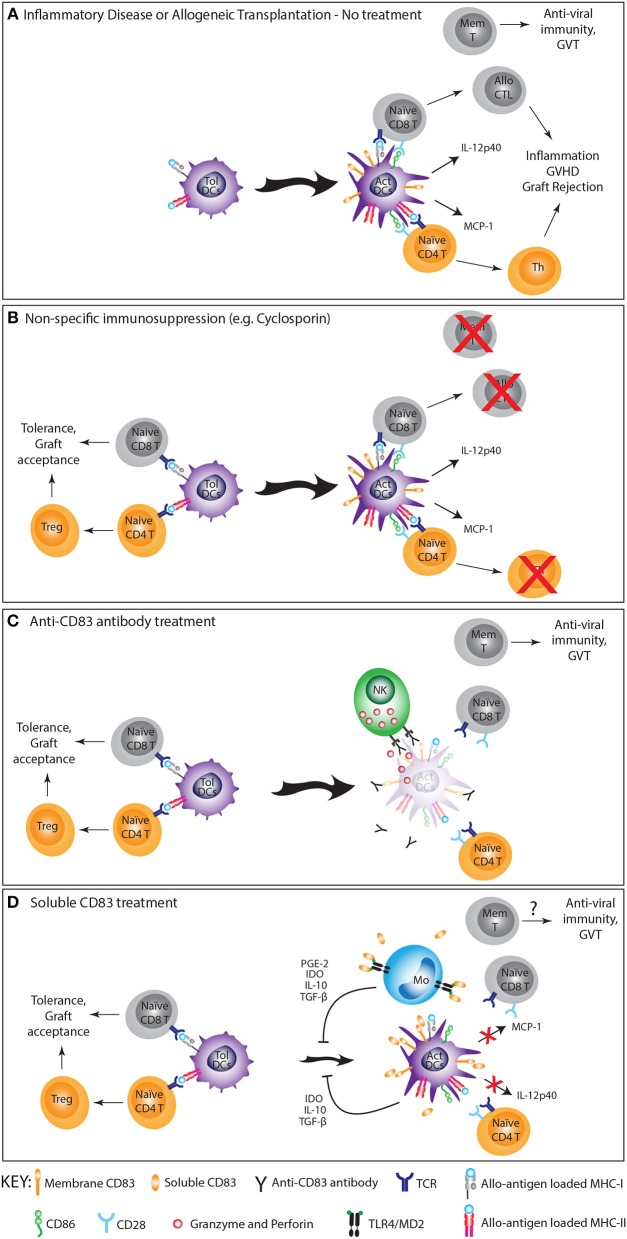
The immunosuppressive effect of anti-CD83 antibodies and rsCD83 in allogeneic transplantation and inflammation. **(A)** Activated (Act) DC stimulate T-cells to initiate a damaging alloimmune/inflammatory response caused by allogeneic transplantation or autoimmunity. **(B)** Conventional treatment with non-specific immune suppressive agents (e.g., cyclosporin) controls inflammation, GVHD and graft rejection at the expense of protective infectious and tumor memory T-cell responses. **(C)** Anti-CD83 mAb deplete activated DC but not tolerogenic DC (Tol-DC) at the initiation of the immune response, preventing T-cell activation leading to graft rejection/inflammation but promoting T-cell tolerance. It also preserves memory (mem) T-cells capable of protective anti-viral and graft-vs.-tumor (GVT) activities. **(D)** rsCD83 induces IL-10, IDO, PGE-2, and TGF-β from monocytes and DC through homotypic and heterotypic ligand binding, promoting Treg generation, inhibiting DC activation and reducing CD83 surface expression on DC, leading to reduced T-cell activation, graft acceptance and tolerance. The effect of rsCD83 on memory T-cell responses has not been investigated.

rsCD83 proteins that exploit the suppressive function of CD83 are demonstrated to be effective in the treatment of various mouse models of autoimmune and inflammatory diseases (Figure 1). Intra-peritoneal injection of rsCD83 was effective in preventing EAE, a model of multiple sclerosis (MS), with a reduction in T-cell cytokines including IFN-γ, IL-2, IL-4, and IL-10 (21). In an inflammatory bowel disease model, rsCD83 prevented the symptoms of colitis by decreasing inflammatory cell infiltration and destruction of colonic architecture through inducing long-term expression of IDO by DC (22). IDO^+^ DC actively diverted T-cell responses toward tolerance (83). Similarly, in an experimental autoimmune uveitis (EAU) model, topical application of rsCD83 showed a protective effect resulting from the induction of tolerogenic IDO^+^ DC, suppressing CD4^+^ T-cell activation in eyes and spleen. rsCD83 application also reduced mCD83 expression by CD83^+^CD3^−^NK1.1^+^ cells that normally infiltrate inflamed eyes. When rsCD83 treated-NK cells were transferred into EAU mice, retinal tissue damage was also relieved (23). In a systemic lupus erythematosus model, rsCD83 significantly delayed onset of pathogenic anti-dsDNA autoantibodies and reduced the concentration of anti-histone IgG autoantibodies compared to the control group (24).

In addition to autoimmunity, sCD83 has potential therapeutic value for preventing solid organ transplant rejection. Treatment with rsCD83 delayed acute cellular rejection of MHC-mismatched skin allografts in mice, significantly reducing the recipient's T-cells capacity to respond (16). In other mouse models, rsCD83 prevented renal allograft rejection and corneal transplantation rejection by inducing IDO and TGF-β (17–19). In a cardiac transplant model, the prolonged allogeneic heart graft and donor specific graft tolerance induced by rsCD83 correlated with a reduction in DC activation markers and allogeneic stimulatory capacity (20). Interestingly, while rsCD83 is immunosuppressive in animal models, elevated levels of natural sCD83 were noticed in various human autoimmune and inflammatory diseases, e.g., in synovial fluid (55) and sera of rheumatoid arthritis patients (54) and MS patient sera (53). The significance of elevated sCD83 in these diseases is not yet understood and may be due to self-regulation of immune system.

### Antibody Targeting of CD83^+^ Cells for Treatment of GVHD

Antibody targeting of CD83 offers the possibility of specifically depleting activated APC capable of stimulating allogeneic T-cells while retaining non-activated APC that impart tolerance and memory T-cells crucial for protective immunity against infection and tumors (Figure 1). This therapeutic strategy was initially trialed in pre-clinical models using a polyclonal rabbit anti-human CD83 antibody (9) and later repeated with the high-affinity human anti-human CD83 IgG1 mAb, 3C12C (10). Both antibodies were adept at mediating antibody-dependent cell cytotoxicity (ADCC) against CD83^+^ expressing cells, particularly activated DC, and by doing so prevented allogeneic T-cell proliferation in mixed leukocyte reactions without affecting memory T-cell reactivity to cytomegalovirus and influenza antigens. A single 125 μg dose of either antibody prevented acute GVHD in a preclinical xenogeneic model where human PBMC were transplanted into SCID mice. This without significantly compromising the donor's overall T-cell and Treg numbers nor memory T-cell responses against viral or tumor antigens. Treatment of non-human primates with up to 10 mg/kg 3C12C was found to have no adverse clinical effects or significantly affect total blood cell counts (12). However, specific reductions were noted in the CD83^+^ populations including CD1c^+^ DC and B-cells. Anti-CD83 antibodies can be effective in other inflammatory settings, as demonstrated in a xenogeneic mouse model of giant cell arteritis (GCA). Treatment of SCID mice with a mouse anti-human CD83 mAb depleted activated DC in GCA-affected human artery grafts, preventing graft infiltration and activation of co-transferred human T-cells (11).

### CD83 as a Therapeutic Target and Biomarker in Cancer

The first malignancy to be reported exhibiting surface CD83 expression was Hodgkin lymphoma (HL) (84). Since then, other tumors have been shown to express CD83, including diffuse large B-cell lymphomas (DLBCL), small cell lung cancer and other lung adenocarcinomas and gastric mucosa-associated lymphoid tissue lymphomas (85–88). In addition, CD83 polymorphisms and mutations have been reported in some cancers. For example, somatic mutations of CD83 with unknown significance have been reported in DLBCL (89, 90) and polymorphisms of CD83 were correlated with prognosis of cervical cancer (91).

HL is a B-cell neoplasm defined by the presence of Hodgkin Reed-Sternberg (HRS) cells. Approximately 30% of patients with advanced disease either relapse or become refractory to chemotherapy and their survival is substantially reduced (92). Recently, we determined that CD83 is expressed on HRS cells (12). In the HL setting, antibody-drug conjugates have advantages over naked antibodies in being able to bypass the suppressive tumor microenvironment that can prevent ADCC (15). We showed CD83 was internalized after antibody engagement and developed 3C12C-monomethyl auristatin E toxin conjugates that were effective in killing CD83^+^ HRS cells (12). This provides impetus for the further investigation of anti-CD83 therapeutics for HL.

HL cells were found to secrete sCD83 that suppressed T-cell proliferation, suggestive of a potential mechanism of immune evasion (12). Blocking sCD83 with anti-CD83 antibodies could mitigate sCD83's effect. Interestingly, serum sCD83 protein is increased in patients with HL as well as other hematological malignancies, including chronic lymphocytic leukemia and mantle cell lymphoma, which correlated with decreased survival and clinical therapeutic response (12–14). These studies raise the possibility that sCD83 could be developed as biomarker for HL and other hematological malignancies and be targeted to enhance immune therapies.

## Concluding Remarks

While recognized as a biomarker for activated APC, greater knowledge of the expression and function of CD83 has given rise to therapeutic strategies that target this molecule or its ligands to suppress inflammatory immune responses. rsCD83 constructs accomplish this by exploiting the regulatory signals induced by CD83 whereas anti-CD83 antibodies act by depleting activated antigen presenting cells that promote inflammatory T-cell responses (Figure 1). Both products have shown great promise for treating inflammatory disease in preclinical models, but some key questions regarding their mechanism of action remain. Determining the significance of differences between mouse and human CD83 (particularly for T-cells) would be important for translating research from animal models into humans. For sCD83, the contribution of homotypic and heterotypic ligand binding and the specific signals induced by these interactions require further elucidation. Also pertinent would be examining whether differences exist between natural sCD83 and the recombinant extracellular sCD83 used in the treatment of disease models. Regarding anti-CD83 antibodies, continued work is required to determine whether depletion of activated DC is the main mode of immune suppression given that targeting CD83 with antibodies has also been shown to suppress DC maturation (62). In addition, it is unclear whether targeting other CD83^+^ cells (e.g., T or B-cells) promotes the efficacy of anti-CD83 treatment. It is of great interest to determine if anti-CD83 antibodies could be therapeutic in other inflammatory settings involving activated DC such as solid organ transplant rejection or autoimmunity. The application of anti-CD83 antibodies or derivatives (e.g., antibody-drug conjugates) are likely to extend to the management of cancer, especially hematological malignancies. The translation of therapeutics targeting CD83 hold great promise as more selective strategies for achieving immunosuppression without significantly compromising protective immunity and have the potential to supersede the broad immunosuppressive drugs currently used to treat inflammatory diseases in the clinic.

## Author Contributions

Paper writing by ZL, W-HH, and PS. Editing by XJ, EA, and GC. Figure drawing by PS and W-HH. DH provided the concept and supervised our CD83 work.

### Conflict of Interest Statement

GC is a Director of DendroCyte which has intellectual property associated with CD83. The remaining authors declare that the research was conducted in the absence of any commercial or financial relationships that could be construed as a potential conflict of interest.

## Abbreviations

ADCC: antibody-dependent cell cytotoxicity
APC: antigen presenting cell/s
DC: dendritic cell/s
DLBCL: diffuse large B-cell lymphomas
EAE: experimental autoimmune encephalomyelitis
EAU: experimental autoimmune uveitis
GCA: giant cell arteritis
GVHD: graft-vs. -host disease
HL: Hodgkin lymphoma
HRS: Hodgkin Reed-Sternberg
HSV-1: Herpes-Simplex virus-1
IDO, indolamine 2: 3-dioxygenase
Ig: immunoglobulin
mAb: monoclonal antibody
mCD83: membrane CD83
MHC-II: MHC class II
MS: multiple sclerosis
NK: natural-killer
rsCD83: recombinant soluble CD83
sCD83: soluble CD83
TEC: thymic epithelial cell/s
TLR: toll-like receptor
Treg: regulatory CD4^+^ T-cell.
